# Risk for shock in people with acute myocardial infarction: a middle-range theory

**DOI:** 10.1590/0034-7167-2024-0504

**Published:** 2025-12-08

**Authors:** Anna Livia de Medeiros Dantas, Richardson Augusto Rosendo da Silva, Dandara Nayara de Azevedo Dantas, Fernanda Beatriz Batista Lima e Silva, Maria Isabel da Conceiação Dias Fernandes, Maria Eduarda Silva do Nascimento, Harlon França de Menezes, Ana Beatriz Pereira

**Affiliations:** I Universidade Federal do Rio Grande do Norte. Natal, Rio Grande do Norte, Brazil; II Hospital Universitário Onofre Lopes. Natal, Rio Grande do Norte, Brazil

**Keywords:** Nursing Theory, Nursing Diagnosis, Myocardial Infarction, Shock, Risk Factors, Teoría de Enfermería, Diagnóstico de Enfermería, Infarto Del Miocardio, Choque, Factores de Riesgo

## Abstract

**Objectives:**

to develop a middle-range theory for the risk for shock in people with acute myocardial infarction.

**Methods:**

theoretical-causal validity of the nursing diagnosis "Risk for shock" in people with acute myocardial infarction, based on a middle-range theory. The study followed six stages: definition of the approach for theory construction; definition of conceptual models; definition of main concepts; development of pictorial diagram; construction of propositions; establishment of causal relationships and evidence for practice.

**Results:**

three essential attributes were identified, 23 clinical antecedents related to the risk for shock, eight theoretical propositions were formulated, and causal relationships were established between the identified stimuli and the essential attributes.

**Conclusions:**

the theory developed established relationships between theory and practice based on the establishment of propositions and causal relationships that allow nurses' diagnostic reasoning.

## RESUMEN

**Objetivos:**

desarrollar una teoría de rango medio para el riesgo de shock en personas con infarto agudo de miocardio.

**Métodos:**

validação teórico-causal del diagnóstico de enfermería "Riesgo de shock" en personas con infarto agudo de miocardio, basado en una teoría de rango medio. El estudio siguió seis etapas: definir el enfoque para la construcação de la teoría; definiação de modelos conceptuales; definiação de conceptos principales; desarrollo del diagrama pictórico; construcação de proposiciones; establecimiento de relaciones causales y evidencia para la práctica.

**Resultados:**

se identificaron tres atributos esenciales, 23 antecedentes clínicos relacionados con el riesgo de choque, se formularon ocho proposiciones teóricas y se establecieron relaciones causales entre los estímulos identificados y los atributos esenciales.

**Conclusiones:**

la teoría desarrollada estableció relaciones entre la teoría y la práctica a partir del establecimiento de proposiciones y relaciones causales que permiten el razonamiento diagnóstico de las enfermeras.

## INTRODUCTION

In recent decades, cardiovascular diseases, specifically ischemic heart disease, have become the leading cause of death in Brazil and worldwide. It is estimated that 19.8 million people will die from cardiovascular diseases in 2022, followed by cancer, chronic respiratory disease and diabetes^(1,2)^.

Patients with myocardial infarction are at imminent risk for losing their life or organ/system function in the human body, progressing to states of shock and multisystem involvement^(3)^.

Shock consists of inadequate perfusion to meet tissue oxygen demand, and can be potentially lethal. It can lead to varying degrees of organic impairment, which requires nurses to have the ability to observe, reason clinically and diagnose. Therefore, it is essential that these professionals know how to identify the risk factors for the occurrence of shock, in order to prevent it in patients with myocardial infarction^(4,5)^.

To this end, the importance of using standardized nursing diagnosis language systems for clinical practice is highlighted, since they offer guidance for selecting interventions capable of producing the desired treatment effects, knowledge of care priorities and care proposals for preventing the unwanted event^(6)^.

The nursing diagnosis (ND) "Risk for shock" is defined by NANDA International (NANDA-I) as "susceptible to an inadequate blood flow to tissues that may lead to cellular dysfunction, which may compromise health". In the context of patients with acute myocardial infarction (AMI), this diagnosis is of utmost importance, since this population is more susceptible to the occurrence of shock^(7)^. However, this diagnosis is quite generalized for the various types of shock, and its latest update was carried out based on studies of patients with septic shock. Therefore, the importance of validating its clinical indicators aimed at patients with ischemic cardiovascular events is highlighted.

The theoretical-causal validation of a ND can be achieved through the development of a middle-range theory (MRT). MRTs have the ability to fill gaps between theory and practice, since they are capable of describing, explaining and predicting phenomena^(8)^.

Therefore, since the aforementioned diagnosis lacks validity studies for this population, in order to direct its definitions and clinical indicators, and better describe the phenomenon, it is important to develop a MRT aimed at the risk for shock in people with myocardial infarction, justifying the performance of this study.

## OBJECTIVES

To develop a MRT for the risk for shock in people with AMI.

## METHODS

### Ethical aspects

As this was a theoretical development study, it did not require approval from the Research Ethics Committee, since there was no direct participation of human beings.

### Study design and period

This is a theoretical development study aimed at the theoretical-causal validity of the ND "Risk for shock" in patients with AMI. For this purpose, an MRT was developed, which was operationalized from an integrative literature review and anchored from the concepts proposed by Roy's Adaptation Model^(9)^. The Preferred Reporting Items for Systematic Reviews and Meta-Analyses (PRISMA) of the Enhancing the QUAlity and Transparency Of health Research network guidelines were followed.

To construct the MRT, the Lopes and Silva^(8)^ methodological framework was used, developed in six stages: definition of the approach for constructing the MRT; definition of conceptual models to be analyzed; definition of key concepts; construction of a pictogram; construction of propositions; and establishment of causal relationships and evidence for practice.

### Study protocol and sample

The construction of an MRT for the risk for shock in people with myocardial infarction was based on the concepts used by Roy in his adaptation model, namely: focal and contextual stimuli, behaviors and the physiological mode (basic need for adaptation). In relation to the physiological mode and the need for oxygenation, in Roy's Adaptation Model, adaptive problems related to hypoxia/shock and inadequate tissue perfusion are described^(9)^.

This mode described by Roy represents the physical response to environmental stimuli with a focus on the regulatory system. In patients with AMI at risk for shock, physiological integrity is directly related to oxygenation needs, as a basic need for a person's adaptive response. The nursing goal is to promote adaptive responses, which are influenced by stimuli, which are elements brought by Roy in his adaptive model^(9)^.

To classify the causal factors of the risk of shock diagnosis into focal, contextual stimuli expressed by Roy in his Adaptation Model, the following criteria were used: focal stimuli represent the main trigger that requires an adaptive response, are related to factors that cause the greatest impact on the behavior pattern and are modifiable with nursing interventions independently. In this study, the risk factors were classified as focal stimuli and, therefore, aspects that require direct and immediate interventions. Contextual stimuli are those that influence the focal stimulus, and were classified in this study as the associated conditions and populations at risk of the ED. Thus, they should be managed together with the multidisciplinary team to support the adaptive process and minimize factors that aggravate the risk of shock.

Residual stimuli are less evident factors, often subjective or unknown, that can influence the adaptation process in a subtle way. In this study, no diagnostic component was compared to residual stimuli.

The main concepts related to the risk of shock were selected from the findings of the integrative review, based on Roy's adaptive model and the components of the NANDA-I diagnosis. In short, the central concepts for the construction of the TMA were extracted. The conceptual definition of the risk of shock in patients with acute myocardial infarction was also constructed, based on the essential attributes identified in the integrative review. The integrative review was carried out through the following steps: problem identification; literature search; data evaluation; data analysis; and data presentation.

The Population, Concept, and Context (PCC) strategy was applied to guide the development of the research question. Thus, the Population listed was patients with myocardial infarction; the Concept was the risk for shock; and the Context is related to the Intensive Care Unit (ICU). Thus, the main guiding question developed was: what are the essential attributes and clinical antecedents of the risk for shock in patients with myocardial infarction in the ICU? Furthermore, to contemplate the research objective, the following subsequent research question was developed: what are the causal relationships between the attributes of risk for shock and clinical antecedents in patients with myocardial infarction in the ICU?.

The searches were carried out by two researchers in September 2024 in the electronic databases Science Direct, PubMed/MEDLINE, Scientific Electronic Library Online (SciELO), Scopus, CINAHL, Embase and Web of Science. The controlled descriptors of Medical Subject Headings (MESH) were used with the following crossings: #1 "Myocardial Infarction" AND "Shock" AND "Risk Factors" AND "intensive care units"; #2 "Myocardial Infarction" AND "Shock" AND "coronary care units".

The findings were analyzed in a double-blind format by two independent researchers, with the help of Rayyan software. A third researcher analyzed the disagreement between the two evaluators to define the final sample. To compose the sample, studies available in full that answered the guiding questions of this study were included. Abstracts, preliminary notes, editorials, secondary studies and letters to the editor were excluded.

**Figure 1:**
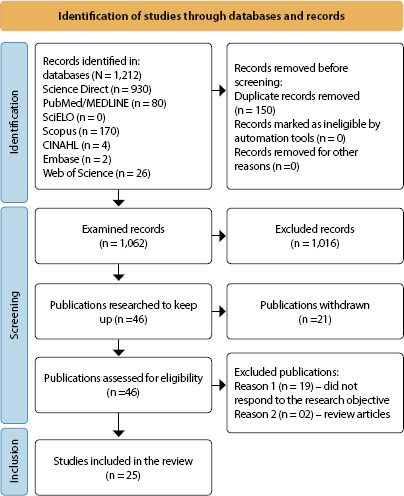
PRISMA diagram referring to database searches for the integrative literature review.

The variables of interest that comprised the data collection instrument were distributed into the following categories: 1) Study characterization (title, authors, place of publication, year of publication, objective, type of study/document, place of development, population in which the study/document was developed); 2) Concept of shock risk (meaning, use of the concept, application of concept, contextual basis, attributes, substitute terms, related concepts, antecedents, operational definitions, associated conditions and populations at risk). For better visualization and synthesis of selected studies, the standard model of methodological presentation of the study was used according to the PRISMA diagram in Figure 1 ^(10)^.

.

### Analysis of results, and statistics

The studies were categorized and the information extracted. The findings were identified as essential attributes and clinical antecedents. An illustrated pictogram was also created, and propositions were drawn up for the relationships between diagnosis and its clinical indicators, and interrelationships were constructed between the stimuli and behaviors related to the risk of shock in patients with AMI. The data were analyzed according to the adopted framework and related literature.

## RESULTS

A total of 1,212 articles were selected from the databases, of which 150 were removed due to duplication. Of the 1,062 articles, 1,016 were excluded after reading the title and abstract. After analysis by the two researchers and the reviewer, 46 articles remained for reading the full text in full. Of the 46 articles, 25 comprised the final sample, since 21 articles did not meet the study objective or were review articles, as shown in the flowchart shown in Figure 1. In relation to study design, 18 were retrospective cohort articles; four were prospective cohort articles; and three were randomized clinical trials.

The MRT was developed based on 25 articles identified in the integrative review. It was possible to extract the main concepts related to the risk for shock in patients with myocardial infarction. In summary, three essential attributes and 23 clinical antecedents were identified, which were subdivided into risk factors, associated conditions and populations at risk.

Reduced capillary filling pressure^(11,12)^, insufficient nutrient supply to the tissues^(13-15)^ and cardiac output inadequate to meet the body's oxygen demand^(12,14)^ were listed as essential attributes. Thus, the risk for shock in people with AMI was defined as "susceptible to an inadequate blood flow to tissues that may lead to cellular dysfunction, which may compromise health".

Clinical history was categorized as proposed by Roy's Adaptation Model, with three risk factors (focal stimuli), 16 associated conditions (contextual stimuli) and three at-risk populations (contextual stimuli), as shown in Chart 1.

**Chart 1:** Clinical stimuli/antecedents of shock risk in patients with acute myocardial infarction, Natal, Rio Grande do Natal, Brazil, 2024.

Risk factors/focal stimuli	Associated conditions/contextual stimuli	Populations at risk/contextual stimuli
Excessive bleeding ^(14)^; insufficient fluid volume^(15)^; non-hemorrhagic fluid loss^(16)^.	Diabetes mellitus^(13,17)^; acute myocardial infarction^(18)^; heart failure^(12)^; early coronary angiography^(19)^; prolonged ischemia time^(20)^; multivessel disease^(20)^; vasopressor drugs^(21)^; electrocardiogram conduction disturbances^(11)^; early transluminal coronary angioplasty^(19)^; ejection fraction less than 35%^(18)^; smoking^(22)^; systemic inflammatory response syndrome^(23)^; inadequate fluid balance^(13)^; inadequate hemodynamic monitoring^(13)^; peripheral arterial disease^(11)^; severe heart disease^(22)^.	Older adults^(13,17)^; individuals with a history of myocardial infarction^(15,17)^; female^(5)^.

**Figure 2:**
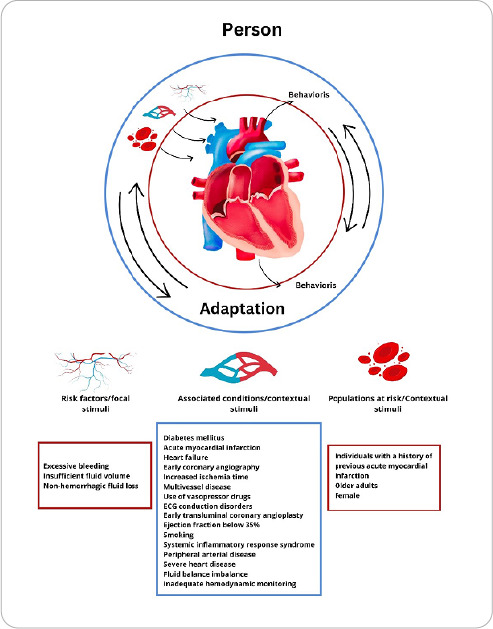
Pictogram of concepts involved in the risk for shock in patients with acute myocardial infarction, Natal, Rio Grande do Norte, Brazil, 2024

The constructed pictogram, as presented in Figure 2, explains the relationships between the concepts involving the risk for shock in patients with AMI. In this regard, to explain the risk for shock in patients with myocardial infarction, it was considered that a person is influenced by several circulatory stimuli, with the heart playing a central role in the physiological response, and these stimuli that can cause the occurrence of ineffective behavior, such as decreased capillary filling pressure, insufficient supply of nutrients to the tissues and decreased cardiac output in the context of the physical-physiological mode of Roy's Adaptation Theory (oxygenation/hypoxia/shock/altered tissue perfusion).

.

In patients with acute myocardial infarction, the core of the problem is the deficit in oxygen supply to the heart muscle, which is why the coronary arteries represent the focal stimuli in the pictogram, since the interruption of blood flow to the coronary arteries has a direct impact, causes myocardial injury and failure in the heart pump, and is responsible for increasing the patient's vulnerability to developing ineffective behavior, which would be low cardiac output and consequent hypoperfusion.

The large and small circulations, expressed in the pictogram, represent contextual stimuli, because although they are not the main problem causing the risk for shock in a heart attack, they enhance and reinforce the development of low cardiac output and hypoperfusion of target organs for the individual. The large circulation ensures oxygenation and nutrition for all cells in the body, and the small circulation is responsible for carrying blood from the heart to the lungs and carrying out the hematosis process.

From the identification of essential attributes, clinical antecedents and preparation of the pictogram, it was possible to interrelate the concepts and develop eight propositions for the diagnosis of risk for shock in patients with myocardial infarction, as follows.

1. Focal and contextual stimuli interfere with the physical-physiological mode of individuals with myocardial infarction, increasing patients' vulnerability to the occurrence of ineffective behavior of reducing cardiac output, capillary filling pressure and insufficient nutritional supply to the tissues;.
2. Focal stimuli such as excessive bleeding, non-hemorrhagic fluid loss and insufficient fluid volume are directly related to increased vulnerability to low cardiac output, capillary refill pressure and insufficient nutritional supply to the tissues. These stimuli are external to individuals and modifiable, and subject to intervention by intensive care team professionals;.
3. Excessive bleeding represents a focal stimulus that directly reduces intravascular volume, compromising perfusion and exacerbating the risk for shock in vulnerable patients;.
4. Insufficient fluid volume contributes to hypovolemia, affecting cardiac preload and increasing the risk for circulatory failure associated with shock;.
5. Non-hemorrhagic fluid losses (such as excessive sweating, vomiting or diarrhea) compromise hemodynamic balance and may contribute to reduced tissue perfusion, increasing the risk for shock;.
6. Contextual stimuli such as chronic diseases, heart failure, ejection fraction below 35%, smoking, systemic inflammatory response syndrome, peripheral arterial disease, multivessel disease, ECG conduction disorders and heart disease are associated with processes intrinsic to the individual. These stimuli interfere with the physical-physiological mode of the patient with myocardial infarction, in such a way as to enhance the occurrence of ineffective behaviors;.
7. Contextual stimuli such as coronary angiography, ischemia time, vasopressor drugs and transluminal coronary angioplasty, inadequate fluid balance and inadequate hemodynamic monitoring are factors external to individuals, relating to clinical judgment and professional conduct. These stimuli can enhance the development of ineffective behavior;.
8. Older adult, female and with a history of previous myocardial infarction are populations at risk for the risk for shock..

## DISCUSSION

The development of MRTs is important for the advancement of nursing knowledge, as it enables the application of theory to practical phenomena in its various areas of activity. In recent years, nurses have shown an interest in developing MRTs to support care in clinical practice. However, there is a gap in knowledge regarding the phenomenon under study, since the theories found in literature focus on cardiovascular rehabilitation and self-care, which reinforces the need for studies focused on critically ill patients with cardiovascular impairment^(24)^.

The mid-range theory Risk of Shock in people with myocardial infarction was developed with the aim of filling the gaps in this field of knowledge, in order to describe the phenomena related to the development of shock in this population and guide the nurse's decision-making in an assertive manner.

To this end, the findings in the literature showed that the key elements that characterize the risk for shock in patients with AMI were reduced capillary filling pressure, insufficient supply of nutrients to the tissues, and inadequate cardiac output to meet the body's oxygen demand. These essential attributes, when influenced by etiological factors and interacting, contribute to reaching the threshold for the occurrence of the diagnosis of shock risk in patients with AMI.

Strict monitoring of fluid balance is essential in patients with myocardial infarction, since inadequate circulatory volume can lead to insufficient tissue perfusion and oxygen supply, and fluid overload can cause heart failure and pulmonary edema. Therefore, studies show that the prognosis of shock is related to the degree of hemodynamic abnormalities, and inadequate fluid balance can increase mortality associated with these complications^(25,26)^.

Hemorrhagic losses were also considered risk factors for the occurrence of shock, according to the findings of this study. Bleeding in critically ill patients with myocardial infarction can occur due to complications arising from the percutaneous procedure, such as bleeding at the insertion site, retroperitoneal bleeding, digestive bleeding, and hematoma. It can also be related to the use of anticoagulant and antiplatelet agents. Hemorrhagic losses reduce circulating volume and consequently cardiac output, increasing the risk of shock^(4,27,28)^.

Myocardial injury of ischemic origin can lead to cardiac pump failure and consequent decrease in cardiac output. Fluid replacement is the first intervention performed in patients with shock, with the aim of promoting an increase in preload and cardiac output. However, fluid administration in patients with myocardial infarction should be performed with caution, since unnecessary fluid infusion is associated with an excessively positive fluid balance, with negative effects on the outcome of critically ill patients, including death^(5,25,29)^.

In addition to bleeding, patients with infarction and risk for shock may face non-hemorrhagic fluid losses, since the mechanism of intravascular volume reduction in patients with infarction and shock occurs because low cardiac output leads to redistribution of blood flow, prioritizing cerebral and coronary flow^(5,29)^.

Chronic conditions such as diabetes mellitus, heart failure and heart disease can favor the occurrence of shock in patients with AMI. They are considered associated conditions, since they are elements that interact with the person, producing physiological responses, but are not directly subject to nurse intervention^(30,31)^. Diabetic patients generally present with sensory and autonomic neuropathy that can mask the ischemic symptoms presented in AMI, which generally delays diagnosis and treatment. In addition, hyperglycemia causes increased platelet aggregation and changes in erythrocyte function, which can lead to the formation of thrombi^(31)^. As for heart disease, these can compromise cardiac performance with a consequent reduction in oxygen supply to the tissues and contribute to the occurrence of shock^(4-33)^.

Multivessel coronary disease is present in up to 50% of patients with myocardial infarction, and the presence of this factor influences the occurrence of new coronary events. Treatment of multivessel lesions is guided according to the presence or absence of shock, since multivessel percutaneous coronary intervention in patients with cardiogenic shock is associated with a significantly higher risk for death^(34,35)^.

Access to transluminal coronary angiography and percutaneous angioplasty are considered essential for reducing complications such as shock, since they determine the degree of arterial impairment, aid in risk classification and promote coronary reperfusion in a less invasive manner. Studies indicate that if performed within the first 90 minutes after the diagnosis of infarction, there is a considerable reduction in the occurrence of complications, especially cardiogenic shock^(36,37)^.

Regarding populations at risk for shock, the literature shows that individuals who are over 60 years of age, with a history of another ischemic cardiovascular event and who are female have a higher risk for developing shock. Older patients who develop myocardial infarction are statistically more affected by cardiogenic shock^(5,36)^.

Patients with a previous history of myocardial infarction are more likely to develop cardiogenic shock in the context of a new ischemic event^(30)^. Although the incidence of myocardial infarction is higher in males, the occurrence of cardiogenic shock in patients with myocardial infarction is higher in females. This is because women present atypical clinical manifestations of acute coronary syndrome, which can delay diagnosis and treatment. In addition, women with acute coronary syndrome tend to be older and have more comorbidities^(5,30,36)^.

### Study limitations

As limitations of this study, it is worth highlighting that the MRT constructed was aimed at patients with myocardial infarction, i.e., for a highly specific population, which may limit the application of the concepts to other populations.

### Contributions to nursing

The MRT "risk for shock in people with AMI" provided theoretical support for the review of the ND "Risk for shock" of the NANDA-I taxonomy with the aim of suggesting risk factors, associated conditions and populations at risk that allow clinical judgment and diagnostic reasoning of intensive care nurses in cardiology. Furthermore, TMA contemplated an approach based on Roy's Adaptation Model, which provides guidelines for nurses to apply the nursing process in their practice.

## CONCLUSIONS

The construction of an MRT achieved the theoretical-causal validity of the ND "Risk for shock" in people with myocardial infarction, as it allowed a better understanding of the factors that interfere with the occurrence of shock in this population. Three essential attributes, 23 clinical antecedents and eight theoretical propositions were identified that allowed establishing the interrelations between concepts.

The study allowed the application of Roy's Adaptation Model, described in a grand theory, as a tool for guiding the nursing process and providing evidence-based foundation for its use in practice. This study also serves as a subsidy for conducting clinical validation research with the aim of testing causal relationships in practice.

## Data Availability

The research data are available only upon request.
