# Supplementary material for: Risk for shock in people with acute myocardial infarction: a middle-range theory
Source: Rev Bras Enferm. 2025 Dec 8;78(4):e20240504. doi: 10.1590/0034-7167-2024-0504 (PMC12716810; doi:10.1590/0034-7167-2024-0504)
Supplement: Translation

# Translation: Risco de choque em pessoas com infarto agudo do miocárdio: Teoria de Médio Alcance

**DOI:** 10.1590/0034-7167-2024-0504pt

**Authors:** Anna Livia de Medeiros Dantas, Richardson Augusto Rosendo da Silva, Dandara Nayara de Azevedo Dantas, Fernanda Beatriz Batista Lima e Silva, Maria Isabel da Conceiação Dias Fernandes, Maria Eduarda Silva do Nascimento, Harlon França de Menezes, Ana Beatriz Pereira

**Affiliations:** I Universidade Federal do Rio Grande do Norte. Natal, Rio Grande do Norte, Brasil; II Hospital Universitário Onofre Lopes. Natal, Rio Grande do Norte, Brasil

**Keywords:** Teoria de Enfermagem, Diagnóstico de Enfermagem, Infarto do Miocárdio, Choque, Fatores de Risco

## Abstract

**Objetivos:**

desenvolver uma teoria de médio alcance para o risco de choque em pessoas com infarto agudo do miocárdio.

**Métodos:**

validação teórico-causal do diagnóstico de enfermagem "Risco de choque" em pessoas com infarto agudo do miocárdio, a partir de uma teoria de médio alcance. O estudo seguiu seis etapas: definiação da abordagem para construação da teoria; definiação dos modelos conceituais; definiação dos conceitos principais; desenvolvimento do diagrama pictórico; construação das proposições; estabelecimento das relações causais e evidências para a prática.

**Resultados:**

foram identificados três atributos essenciais, 23 antecedentes clínicos relacionados ao risco de choque, formuladas oito proposições teóricas e estabelecidas relações causais entre os estímulos identificados e os atributos essenciais.

**Conclusões:**

a teoria desenvolvida estabeleceu relações entre teoria e prática a partir do estabelecimento de proposições e relações causais que permitem o raciocínio diagnóstico dos enfermeiros.

## INTRODUÇÃO

Nas últimas décadas, as doenças cardiovasculares, especificamente a doença isquêmica do coração, se tornaram as principais causas de morte no Brasil e no mundo. Estima-se que 19,8 milhões de pessoas morreram de doenças cardiovasculares em 2022, seguidas de câncer, doença respiratória crônica e diabetes^(1,2)^.

O paciente com infarto do miocárdio encontra-se em risco iminente de perder a vida ou funação de órgão/sistema do corpo humano, evoluindo com estados de choque e comprometimento multissistêmico^(3)^.

O choque consiste na inadequada perfusão para atender à demanda de oxigênio tecidual, podendo tornar-se potencialmente letal. Pode levar a diferentes graus de comprometimento orgânico, que exige do enfermeiro a capacidade de observação, raciocínio clínico e diagnóstico. Portanto, é essencial que esse profissional saiba identificar os fatores de risco para ocorrência do choque, com a finalidade de preveni-lo em pacientes com infarto do miocárdio^(4,5)^.

Para tanto, ressalta-se a importância da utilização de sistemas padronizados de linguagem dos diagnósticos de enfermagem para a prática clínica, uma vez que oferecem orientação para seleação de intervenções capazes de produzir os efeitos desejados do tratamento, conhecimento de prioridades de atendimento e proposições de cuidados para a prevenação do evento indesejado^(6)^.

O diagnóstico de enfermagem (DE) "Risco de choque" é definido pela NANDA Internacional (NANDA-I) como "susceptibilidade a uma condiação manifestada pela falha na perfusão ou oxigenação de órgãos vitais". No contexto do paciente com infarto agudo do miocárdio (IAM), esse diagnóstico é de suma importância, visto que essa população está mais suscetível à ocorrência do choque^(7)^. Todavia, esse diagnóstico é bastante generalizado para os diversos tipos de choque, e sua última atualização foi realizada com base em estudos de pacientes com choque séptico. Para tanto, ressalta-se a importância da validação de seus indicadores clínicos direcionados para o paciente com evento cardiovascular isquêmico.

A validação teórico-causal de um DE pode ser realizada por meio do desenvolvimento de uma teoria de médio alcance (TMA). As TMAs têm a capacidade de preencher lacunas existentes entre a teoria e a prática, visto que são capazes de descrever, explicar e prever fenômenos^(8)^.

Assim sendo, uma vez que o referido diagnóstico carece de estudos de validação para essa população, a fim de direcionar suas definições e indicadores clínicos, e descrever melhor o fenômeno, ressalta-se a importância do desenvolvimento de uma TMA direcionada para o risco de choque em pessoas com infarto do miocárdio, justificando a realização do presente estudo.

## OBJETIVOS

Desenvolver uma TMA para o risco de choque em pessoas com IAM.

## MÉTODOS

### Aspectos éticos

Por tratar-se de estudo de desenvolvimento teórico, não necessitou de aprovação do Comitê de Ética em Pesquisa, uma vez que não houve a participação de seres humanos de forma direta.

### Desenho e período de estudo

Estudo de desenvolvimento teórico, orientado para a validade teórico-causal do DE "Risco de choque" em pacientes com IAM. Para isso, desenvolveu-se uma TMA, a qual foi operacionalizada a partir de uma revisão integrativa da literatura e ancorada a partir dos conceitos propostos pelo Modelo de Adaptação de Roy^(9)^. Seguiram-se as diretrizes do instrumento *Preferred Reporting Items for Systematic Reviews and Meta-Analyses* (PRISMA) da *Enhancing the QUAlity and Transparency Of health Research network*.

Para a construação da TMA, utilizou-se o referencial metodológico de Lopes e Silva^(8)^, desenvolvido em seis etapas: definiação da abordagem para construação do TMA; definiação dos modelos conceituais a serem analisados; definiação de conceitos-chave; construação de um pictograma; construação de proposições; e estabelecimento de relações causais e evidências para a prática.

### Protocolo de estudo e amostra

A construação da TMA para o risco de choque em pessoas com infarto do miocárdio baseou-se nos conceitos utilizados por Roy em seu modelo da adaptação, a saber: nos estímulos focais e contextuais, nos comportamentos e no modo fisiológico (necessidade básica adaptação). Em relação ao modo fisiológico e a necessidade de oxigenação, do Modelo de Adaptação de Roy, estão descritos problemas adaptativos relacionados à hipóxia/choque e perfusão inadequados dos tecidos^(9)^.

Esse modo descrito por Roy representa a resposta física aos estímulos ambientais com enfoque no sistema regulador. Em pacientes com IAM com risco de choque, a integridade fisiológica está diretamente relacionada à s necessidades de oxigenação, como necessidade básica para a resposta adaptativa da pessoa. A meta de enfermagem é promover as respostas adaptativas, que sofrem influência dos estímulos, que são elementos trazidos por Roy em seu modelo adaptativo^(9)^.

Para a classificação dos fatores causais do diagnóstico risco de choque em estímulos focais, contextuais expressos por Roy, em seu Modelo de Adaptação, utilizaram-se os seguintes critérios: os estímulos focais representam o principal gatilho que exige uma resposta adaptativa, estão relacionados a fatores que causam maior impacto no padrão de comportamento e que são modificáveis com as intervenções de enfermagem de forma independente. Neste estudo, os fatores de risco foram classificados como estímulos focais e, portanto, aspectos que demandam intervenções diretas e imediatas. Os estímulos contextuais são aqueles que influenciam o estímulo focal, e que foram classificados neste estudo como as condições associadas e populações em risco do DE. Desse modo, devem então ser manejadas junto a equipe multiprofissionais para apoiar o processo adaptativo e minimizar fatores que agravam o risco de choque.

Os estímulos residuais são fatores menos evidentes, frequentemente subjetivos ou desconhecidos, que podem influenciar no processo de adaptação de forma sutil. Neste estudo, nenhum componente diagnóstico foi comparado aos estímulos residuais.

Foram selecionados os principais conceitos relacionados ao risco de choque a partir dos achados da revisão integrativa, com base no modelo adaptativo de Roy e nos componentes do diagnóstico da NANDA-I. Em suma, foram extraídos os conceitos centrais para a construação da TMA. Também foi construída a definiação conceitual do risco de choque em pacientes com infarto agudo do miocárdio, a partir dos atributos essenciais identificados na revisão integrativa. A revisão integrativa foi realizada por meio das seguintes etapas: identificação do problema; pesquisa na literatura; avaliação dos dados; análise de dados; e apresentação dos dados.

Aplicou-se a estratégia População, Conceito e Contexto (PCC) para nortear a elaboração da questão de pesquisa. Assim, a População elencada foi de pacientes com infarto do miocárdio; o Conceito foi o risco de choque; e o Contexto está relacionado à Unidade de Terapia Intensiva (UTI). Dessa forma, a questão norteadora principal elaborada foi: quais são os atributos essenciais e antecedentes clínicos do risco de choque em pacientes com infarto do miocárdio na UTI? Ademais, para contemplar o objetivo da pesquisa, foi elaborada a seguinte questão de pesquisa subsequente: quais são as relações causais entre os atributos do risco de choque e os antecedentes clínicos em pacientes com infarto do miocárdio na UTI?.

As buscas foram realizadas por dois pesquisadores, no mês de setembro de 2024, nas bases de dados eletrônicas *Science Direct*, PubMed/MEDLINE, Scientific Electronic Library Online (SciELO), Scopus, CINAHL, Embase e *Web of Science*. Foram utilizados os descritores controlados do *Medical Subject Headings* (MESH) com os seguintes cruzamentos: #1 "*Myocardial Infarction" AND "Shock" AND "Risk Factors" AND "intensive care units"; #2 "Myocardial Infarction" AND "Shock" AND "coronary care units"*.

Os achados foram analisados no formato duplo cego, por dois pesquisadores independentes, com o auxílio do *software* Rayyan. Um terceiro pesquisador analisou a discordância entre os dois avaliadores para definir a amostra final. Para compor a amostra, foram incluídos estudos disponíveis na íntegra que responderam à s questões norteadoras deste estudo. Foram excluídos resumos, notas prévias, editoriais, estudos secundários e cartas ao editor.

As variáveis de interesse que compuseram o instrumento de coleta de dados foram distribuídas nas seguintes categorias: 1) Caracterização dos estudos (título, autores, local de publicação, ano de publicação, objetivo, tipo do estudo/documento, local de desenvolvimento, população em que o estudo/documento foi desenvolvido); 2) Conceito de risco de choque (significado, uso do conceito, aplicação do conceito, base contextual, atributos, termos substitutos, conceitos relacionados, antecedentes, definições operacionais, condições associadas e populações em risco). Para melhor visualização e síntese dos estudos selecionados, utilizou-se o modelo padrão de apresentação metodológico do estudo conforme o diagrama PRISMA na Figura 1 ^(10)^.

**Figura 1:** Diagrama PRISMA referente à s buscas nas bases de dados para a revisão integrativa da literatura.

.

### Análise dos resultados e estatísticas

Os estudos foram categorizados, e as informações, extraídas. Os achados foram identificados como atributos essenciais e antecedentes clínicos. Foi realizado também um pictograma ilustrado, sendo elaboradas proposições para as relações entre o diagnóstico e seus indicadores clínicos e construídas inter-relações entre os estímulos e comportamentos relacionados ao risco de choque em pacientes com IAM. Os dados foram analisados conforme o referencial adotado e literatura correlata.

## RESULTADOS

Foram selecionados 1.212 artigos nas bases de dados, dos quais 150 foram removidos devido à duplicidade. Dos 1.062 artigos, foram excluídos 1.016 após a leitura do título e resumo. Após análise das duas pesquisadoras e do revisor, permaneceram 46 artigos para a leitura do texto completo na íntegra. Dos 46 artigos, 25 compuseram a amostra final, uma vez que 21 artigos não atendiam ao objeto de estudo ou eram artigos de revisão, conforme fluxograma apresentado na Figura 1. No que tange ao desenho dos estudos, 18 eram artigos de coorte retrospectiva; quatro coorte prospectiva; e três eram ensaios clínicos randomizados.

A TMA foi desenvolvida com base em 25 artigos identificados na revisão integrativa. Foi possível extrair os principais conceitos relacionados ao risco de choque em pacientes com infarto do miocárdio. Em suma, foram identificados três atributos essenciais e 23 antecedentes clínicos, que foram subdivididos em fatores de risco, condições associadas e populações em risco.

Reduação da pressão de enchimento capilar^(11,12)^, aporte insuficiente de nutrientes para os tecidos^(13-15)^ e débito cardíaco inadequado para atender à demanda de oxigênio do corpo^(12,14)^ foram listados como atributos essenciais. Assim, o risco de choque em pessoas com IAM foi definido como "susceptibilidade à falência circulatória resultante da oferta insuficiente de oxigênio para os tecidos".

Os antecedentes clínicos foram categorizados conforme proposto pelo Modelo de Adaptação de Roy, sendo três fatores de risco (estímulos focais), 16 condições associadas (estímulos contextuais) e três populações de risco (estímulos contextuais), conforme o Quadro 1.

**Quadro 1:** Estímulos/antecedentes clínicos do risco de choque em pacientes com infarto agudo do miocárdio, Natal, Rio Grande do Norte, Brasil, 2024.

Fatores de risco/estímulos focais	Condições associadas/estímulos contextuais	Populações em risco/estímulos contextuais
Sangramento excessivo ^(14)^; volume de líquido insuficiente^(15)^; perdas não hemorrágicas de líquidos^(16)^.	Diabetes mellitus^(13,17)^; infarto agudo do miocárdio^(18)^; insuficiência cardíaca^(12)^; angiografia coronariana precoce^(19)^; tempo de isquemia elevado^(20)^; doença multiarterial^(20)^; fármacos vasopressores^(21)^; distúrbios na conduação do eletrocardiograma^(11)^; angioplastia transluminal coronariana precoce^(19)^; fração de ejeação inferior a 35%^(18)^; tabagismo^(22)^; síndrome da resposta inflamatória sistêmica^(23)^; balanço hídrico inadequado^(13)^; monitoramento hemodinâmico inadequado^(13)^; doença arterial periférica^(11)^; cardiopatias graves^(22)^.	Idosos^(13,17)^; indivíduos com história de infarto do miocárdio^(15,17)^; sexo feminino^(5)^.

O pictograma construído, conforme apresentado na Figura 2, explica as relações entre os conceitos envolvendo o risco de choque em pacientes com IAM. Nesse sentido, para explicar o risco de choque em pacientes com infarto do miocárdio, considerou-se que a pessoa é influenciada por diversos estímulos circulatórios, com o coração desempenhando um papel central na resposta fisiológica, e esses estímulos que podem provocar a ocorrência de um comportamento ineficaz, como a diminuiação do da pressão de enchimento capilar, aporte insuficiente de nutrientes para o tecidos e débito cardíaco diminuído no contexto do modo físico-fisiológico da Teoria de Adaptação de Roy (oxigenação/hipóxia/choque/perfusão alterada do tecido).

**Figura 2:** Pictograma dos conceitos envolvidos no risco de choque em pacientes com infarto agudo do miocárdio, Natal, Rio Grande do Norte, Brasil, 2024.

.

No paciente com infarto agudo do miocárdio o cerne central do problema é o déficit de suprimento de oxigênio para o músculo cardíaco, por isso as artérias coronárias estão representando os estímulos focais no pictograma, uma vez que a interrupação do fluxo sanguíneo para as coronárias exerce impacto direto, provoca lesão miocárdica e falha na bomba cardíaca, e são responsáveis por aumentar a vulnerabilidade do paciente para desenvolver o comportamento ineficaz, que seria o baixo débito cardíaco e consequente hipoperfusão.

A grande e a pequena circulação, expressas no pictograma, representam os estímulos contextuais, pois apesar de não serem o principal problema causador do risco de choque no infarto, mas potencializam e reforçam o desenvolvimento do comportamento de baixo débito cardíaco e hipoperfusão de órgãos-alvo para o indivíduo. A grande circulação garante a oxigenação e nutriação para todas as células do corpo. E a pequena circulação é responsável por levar o sangue do coração para os pulmões e realizar o processo de hematose.

A partir da identificação dos atributos essenciais, antecedentes clínicos e elaboração do pictograma, foi possível inter-relacionar os conceitos e elaborar oito proposições para o diagnóstico de risco de choque em pacientes com infarto do miocárdio, conforme segue abaixo.

1. Os estímulos focais e contextuais interferem no modo físico-fisiológico do indivíduo com infarto do miocárdio, aumentando a vulnerabilidade do paciente para a ocorrência do comportamento ineficaz de reduação do débito cardíaco, pressão de enchimento capilar e aporte nutricional insuficiente para os tecidos;.
2. Os estímulos focais comosangramento excessivo, perdas não hemorrágicas de líquidos e volume de líquidos insuficiente se relacionam diretamente com o aumento da vulnerabilidade ao comportamento de baixo débito cardíaco pressão de enchimento capilar e aporte nutricional insuficiente para os tecidos. Esses estímulos são externos ao indivíduo e modificáveis, e passíveis de intervenções dos profissionais da equipe de cuidados intensivos;.
3. O sangramento excessivo representa um estímulo focal que reduz diretamente o volume intravascular, comprometendo a perfusão e exacerbando o risco de choque em pacientes vulneráveis;.
4. O volume de líquido insuficiente contribui para a hipovolemia, afetando a pré-carga cardíaca e aumentando o risco de insuficiência circulatória associada ao choque;.
5. As perdas não hemorrágicas de líquidos (como sudorese excessiva, vômitos ou diarreia) comprometem o equilíbrio hemodinâmico e podem contribuir para a reduação da perfusão tecidual, aumentando o risco de choque;.
6. Os estímulos contextuais como doenças crônicas, insuficiência cardíaca, fração de ejeação inferior a 35%, tabagismo, síndrome da resposta inflamatória sistêmica, doença arterial periférica, doença multiarterial, distúrbios na conduação do ECG e cardiopatias estão associados aos processos intrínsecos ao indivíduo. Esses estímulos interferem no modo físico-fisiológico do paciente com infarto do miocárdio, de forma a potencializar a ocorrência dos comportamentos ineficazes;.
7. Os estímulos contextuais angiografia coronariana, tempo de isquemia, fármacos vasopressores e angioplastia transluminal coronariana, balanço hídrico inadequado e monitoramento hemodinâmico inadequado são fatores externos ao indivíduo, relacionando-se ao julgamento clínico e condutas profissionais. Esses estímulos podem potencializar o desenvolvimento do comportamento ineficaz;.
8. Idosos, do sexo feminino e com histórico de infarto do miocárdio prévio são populações em risco para o risco de choque..

## DISCUSSÃO

O desenvolvimento de TMAs é importante para o avanço do conhecimento da enfermagem, pois possibilita as aplicações da teoria em fenômenos da prática em suas diversas áreas de atuação. Nos últimos anos, percebe-se o interesse dos enfermeiros em desenvolverem TMAs para subsidiar o cuidado na prática clínica. Todavia, verifica-se uma lacuna do conhecimento com relação ao fenômeno em estudo, já que as teorias encontradas na literatura têm enfoque na reabilitação e autocuidado cardiovascular, o que reforça a necessidade de estudos voltados para o paciente crítico com comprometimento cardiovascular^(24)^.

A teoria de médio alcance Risco de Choque em pessoas com infarto do miocárdio foi desenvolvida com o intuito de preencher as lacunas existentes nesse campo de conhecimento, a fim de descrever os fenômenos relacionados ao desenvolvimento do choque nessa população e orientar a tomada de decisão do enfermeiro de forma assertiva.

Para tanto, os achados na literatura evidenciaram que os elementos-chave que caracterizam o risco de choque em paciente com IAM foram reduação da pressão de enchimento capilar, aporte insuficiente de nutrientes para os tecidos e débito cardíaco inadequado para atender à demanda de oxigênio do corpo. Esses atributos essenciais, ao sofrerem influência dos fatores etiológicos e interagirem, contribuem para o alcance do limiar para a ocorrência do diagnóstico do risco de choque em pacientes com IAM.

O monitoramento do balanço hídrico rigoroso é imprescindível nos pacientes com infarto do miocárdio, visto que o volume circulatório inadequado pode levar a perfusão tecidual e oferta de oxigênio insuficientes, assim como a sobrecarga de fluidos pode provocar insuficiência cardíaca e edema pulmonar. Portanto, estudos demonstram que o prognóstico do choque está relacionado ao grau de anormalidades hemodinâmicas e o balanço hídrico inadequado pode aumentar a mortalidade associada a essas complicações^(25,26)^.

As perdas hemorrágicas também foram consideradas fatores de risco para a ocorrência do choque, de acordo com os achados deste estudo. O sangramento no paciente crítico com infarto do miocárdio pode ocorrer devido complicações decorrentes do procedimento percutâneo, como sangramento no local de inseração, sangramento retroperitoneal, digestivo e hematoma. Pode ainda estar relacionado ao uso de agentes anticoagulantes e antiplaquetários. As perdas hemorrágicas reduzem o volume circulante, consequentemente o débito cardíaco, potencializando o risco de choque^(4,27,28)^.

A lesão miocárdica de origem isquêmica pode levar à falha na bomba cardíaca e consequente diminuiação do débito cardíaco. A reposiação de fluidos é a primeira intervenação realizada no paciente com choque, com o objetivo de promover aumento da pré-carga e do débito cardíaco. No entanto, a administração de fluidos nos pacientes com infarto do miocárdio deve ser realizada com cautela, visto que a infusão desnecessária de fluidos está associada a balanço hídrico excessivamente positivo, com efeitos negativos no desfecho de pacientes críticos, incluindo óbito^(5,25,29)^.

Além do sangramento, o paciente com infarto e risco de choque pode enfrentar perdas não hemorrágicas de líquidos, visto que o mecanismo de reduação de volume intravascular em pacientes com infarto e choque ocorre porque o baixo débito cardíaco leva à redistribuiação do fluxo sanguíneo, priorizando o fluxo cerebral e coronariano^(5,29)^.

Condições crônicas como diabetes mellitus, insuficiência cardíaca e cardiopatias podem favorecer a ocorrência do choque em pacientes com IAM. São consideradas condições associadas, visto que são elementos que interagem com a pessoa, produzindo respostas fisiológicas, porém não estão diretamente passíveis da intervenação do enfermeiro^(30,31)^. Geralmente, pacientes diabéticos apresentam quadro de neuropatia sensitiva e autonômica que pode mascarar os sintomas isquêmicos apresentados no IAM, o que geralmente retarda o diagnóstico e tratamento. Além disso, a hiperglicemia provoca o aumento da agregação plaquetária e alteração da funação eritrocitária, que podem levar à formação de trombos^(31)^. Quanto à s cardiopatias, essas podem comprometer o desempenho cardíaco com consequente reduação da oferta de oxigênio aos tecidos e contribuir para a ocorrência do choque^(4-33)^.

A doença coronária multiarterial está presente em até 50% dos pacientes com infarto do miocárdio, e a presença desse fator influencia a ocorrência de novos eventos coronarianos. O tratamento das lesões multiarteriais é orientado de acordo com a presença ou ausência do choque, visto que a intervenação coronária percutânea multiarterial em pacientes com choque cardiogênico está associada a um risco significativamente maior de morte^(34,35)^.

O acesso à angiografia transluminal coronariana e a angioplastia percutânea são considerados imprescindíveis para a reduação de complicações como o choque, visto que determinam o grau de comprometimento das artérias, auxiliam na classificação de risco e promove a reperfusão coronariana de forma menos invasiva. Estudos apontam que se realizada nos primeiros 90 minutos após o diagnóstico do infarto, ocorre a reduação considerável da ocorrência de complicações, principalmente o choque cardiogênico^(36,37)^.

Em relação à s populações em risco para o choque, a literatura demonstra que os indivíduos que têm idade superior a 60 anos, com histórico de outro evento isquêmico cardiovascular e do sexo feminino têm um risco maior de apresentar o choque. Pacientes idosos que desenvolvem infarto do miocárdio estatisticamente são mais acometidos pelo choque cardiogênico^(5,36)^.

Pacientes com história prévia de infarto do miocárdio têm maior probabilidade de desenvolver o choque cardiogênico no contexto de um novo evento isquêmico^(30)^. Apesar da incidência de o infarto do miocárdio ser maior nas pessoas do sexo masculino, a ocorrência do choque cardiogênico em paciente com infarto do miocárdio é mais elevada nas pessoas do sexto feminino. Isso ocorre porque as mulheres apresentam manifestações clínicas atípicas da síndrome coronariana aguda, o que pode retardar o diagnóstico e o tratamento. Além disso, mulheres acometidas com síndrome coronariana aguda tendem a ser mais idosas e apresentam mais comorbidades^(5,30,36)^.

### Limitações do estudo

Como limitações deste estudo, destaca-se que a TMA construída foi voltada para pacientes com infarto do miocárdio, isto é, para uma população altamente específica, o que pode limitar a aplicação dos conceitos para outras populações.

### Contribuições para a área da enfermagem

A TMA "risco de choque em pessoas com IAM" forneceu substrato teórico para a revisão do DE "Risco de choque" da taxonomia da NANDA-I com o intuito de sugerir fatores de risco, condições associadas e populações em risco que permitem o julgamento clínico e raciocínio diagnóstico dos enfermeiros de cuidados intensivos em cardiologia. Ademais, a TMA contemplou uma abordagem fundamentada no Modelo de Adaptação de Roy, que fornece diretrizes para o enfermeiro aplicar o processo de enfermagem em sua prática.

## CONCLUSÕES

A construação da TMA concretizou a validação teórico-causal do DE "Risco de choque" em pessoas com infarto do miocárdio, na medida em que permitiu melhor compreensão sobre os fatores que interferem na ocorrência do choque nessa população. Foram identificados três atributos essenciais, 23 antecedentes clínicos e oito proposições teóricas que permitiram estabelecer as inter-relações entre os conceitos.

O estudo permitiu a aplicação do modelo de Adaptação de Roy, descrito em uma grande Teoria, como ferramenta para orientação do processo de enfermagem e fundamentação a partir de evidências para seu uso na prática. Este estudo serve ainda de subsídio para a realização de pesquisas de validação clínica com o intuito de testar as relações de causalidade na prática.

## Data Availability

Os dados de pesquisa só estão disponíveis mediante solicitação.
